# Supplementary material for: Biocompatibility of α-Al2O3 Ceramic Substrates with Human Neural Precursor Cells
Source: J Funct Biomater. 2020 Sep 16;11(3):65. doi: 10.3390/jfb11030065 (PMC7563382; doi:10.3390/jfb11030065)
Supplement: Supplementary file 1 [file jfb-11-00065-s001.pdf]

Journal of
*Functional
Biomaterials*

Supplementary Materials of

Article

Biocompatibility of α-Al_2O_3 Ceramic Substrates with Human Neural Precursor Cells

Akrivi Asimakopoulou, Ioannis Gkekas, Georgia Kastrinaki, Alessandro Prigione, Vasileios T. Zaspalis and Spyros Petrakis

The FTIR spectra of cA and ncA samples were measured with Jasco spectrometer (Jasco FTIR-6700, Tokyo, Japan). Seventy scans were collected for each measurement in the spectral range of 4000–400 cm^{-1} with a resolution of 4 cm^{-1}. The ceramic discs were pestled to powder and mixed with spectroscopic grade KBr into pellet form.

Supplementary Figure S1 shows two spectra; the cA spectrum (yellow color) exhibits distinct broad peak at 3404 cm^{-1} characteristic for hydroxyl group, while a shorter peak at 1640 cm^{-1} is attributed to –H–OH.

The peaks at 450, 595 and 670 cm^{-1} can be assigned to the Al-O stretching mode of the octahedral structure of the a-Al_2O_3, while the 1070 cm^{-1} is assigned to the Al–O–H symmetric bending [1]. The ncA structure (blue color) shows lower peaks for the –OH related bonds and peaks at the 400–1000 cm^{-1} region related to the bohemite structure.

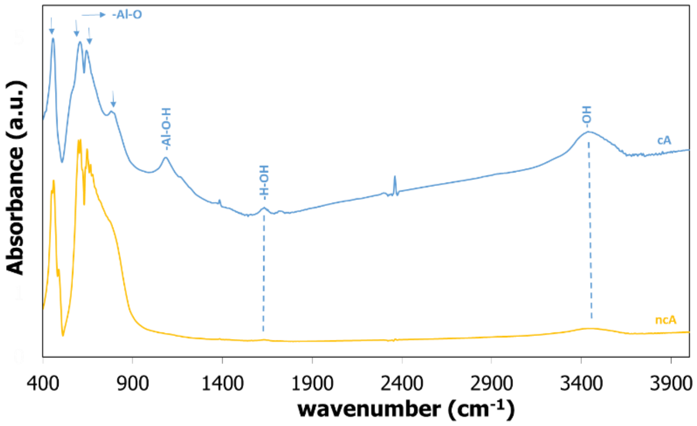

Figure S1. FTIR spectra of cA (blue color) and ncA (yellow color) depicting the characteristic peaks for hydroxyl groups.

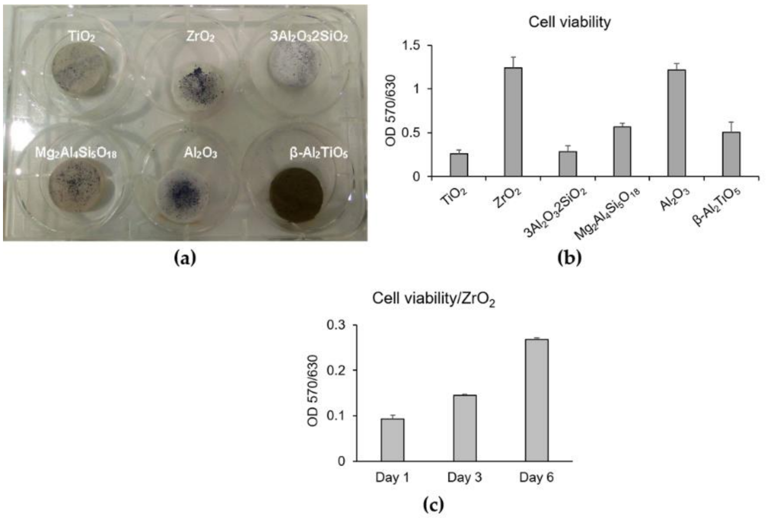

Figure S2. (**a**,**b**) Representative image and quantification of the viability of NPCs seeded on various ceramic substrates. Blue color in (**a**) indicates insoluble formazan crystals formed by viable cells on the surface of each disc. (**c**) Viability of cells cultured for up to 6 days on matrigel-coated ZrO_2 discs.

Supplementary references.

1. Boumaza, A.; Favaro, L.; Lédion, J.; Sattonnay, G.; Brubach, J.B.; Berthet, P.; Huntz, A.M.; Roy, P.; Tétot, R. Transition alumina phases induced by heat treatment of boehmite: An X-ray diffraction and infrared spectroscopy study. *J. Solid State Chem.* **2009**, *182*, 1171–1176, doi.org/10.1016/j.jssc.2009.02.006.
